# The Impact of Conservation Management on the Community Composition of Multiple Organism Groups in Eutrophic Interconnected Man-Made Ponds

**DOI:** 10.1371/journal.pone.0139371

**Published:** 2015-09-30

**Authors:** Pieter Lemmens, Joachim Mergeay, Jeroen Van Wichelen, Luc De Meester, Steven A. J. Declerck

**Affiliations:** 1 Laboratory of Aquatic Ecology, Evolution and Conservation, KU Leuven, Leuven, Belgium; 2 Research Institute for Nature and Forest, Geraardsbergen, Belgium; 3 Laboratory of Protistology and Aquatic Ecology, Ghent University, Ghent, Belgium; 4 Department of Aquatic Ecology, Netherlands Institute of Ecology (NIOO-KNAW), Wageningen, The Netherlands; Estación Biológica de Doñana, CSIC, SPAIN

## Abstract

Ponds throughout the world are subjected to a variety of management measures for purposes of biodiversity conservation. Current conservation efforts typically comprise a combination of multiple measures that directly and indirectly impact a wide range of organism groups. Knowledge of the relative impact of individual measures on different taxonomic groups is important for the development of effective conservation programs. We conducted a field study of 28 man-made ponds, representing four management types differing in the frequency of periodic pond drainage and the intensity of fish stock management. We disentangled the relative importance of direct and indirect effects of pond management measures on the community composition of phytoplankton, zooplankton, aquatic macro-invertebrates, submerged and emergent vascular plants. With the exception of phytoplankton, pond management had strong effects on the community composition of all investigated biota. Whether management affected communities directly or indirectly through its impact on fish communities or local environmental conditions in the pond varied between organism groups. Overall, the impact of pond drainage regime and fish community characteristics on the community composition of target organism groups were more important than local environmental conditions. The majority of taxa were negatively associated with fish density, whereas multiple emergent plant species and several taxa of aquatic macro-invertebrates were positively affected by increased drainage frequency. The effects of fish community and drainage tended to be largely independent. The present study indicates that pond drainage is an important element for biodiversity conservation in eutrophicated shallow and interconnected man-made ponds.

## Introduction

Ponds are increasingly recognized for their high contribution to regional biodiversity [1–4] and the provisioning of vital ecosystem services [5–9]. However, increased human impact, such as nutrient loading, overstocking with fish and reduced water level fluctuation, have resulted in a worldwide deterioration of pond habitats [10, 11] and the local and regional loss of species [5, 12, 13]. Restoration and conservation measures are therefore increasingly applied in efforts to restore pond habitats [14].

A current challenge in conservation biology is the development of effective management programs that maintain and enhance biodiversity in anthropogenic landscapes. This is particularly the case for man-made pond complexes in Western and Central Europe. Many of these systems have high conservation value but are increasingly threatened by the intensification of fish farming [10, 15, 16].

**Fig 1:**
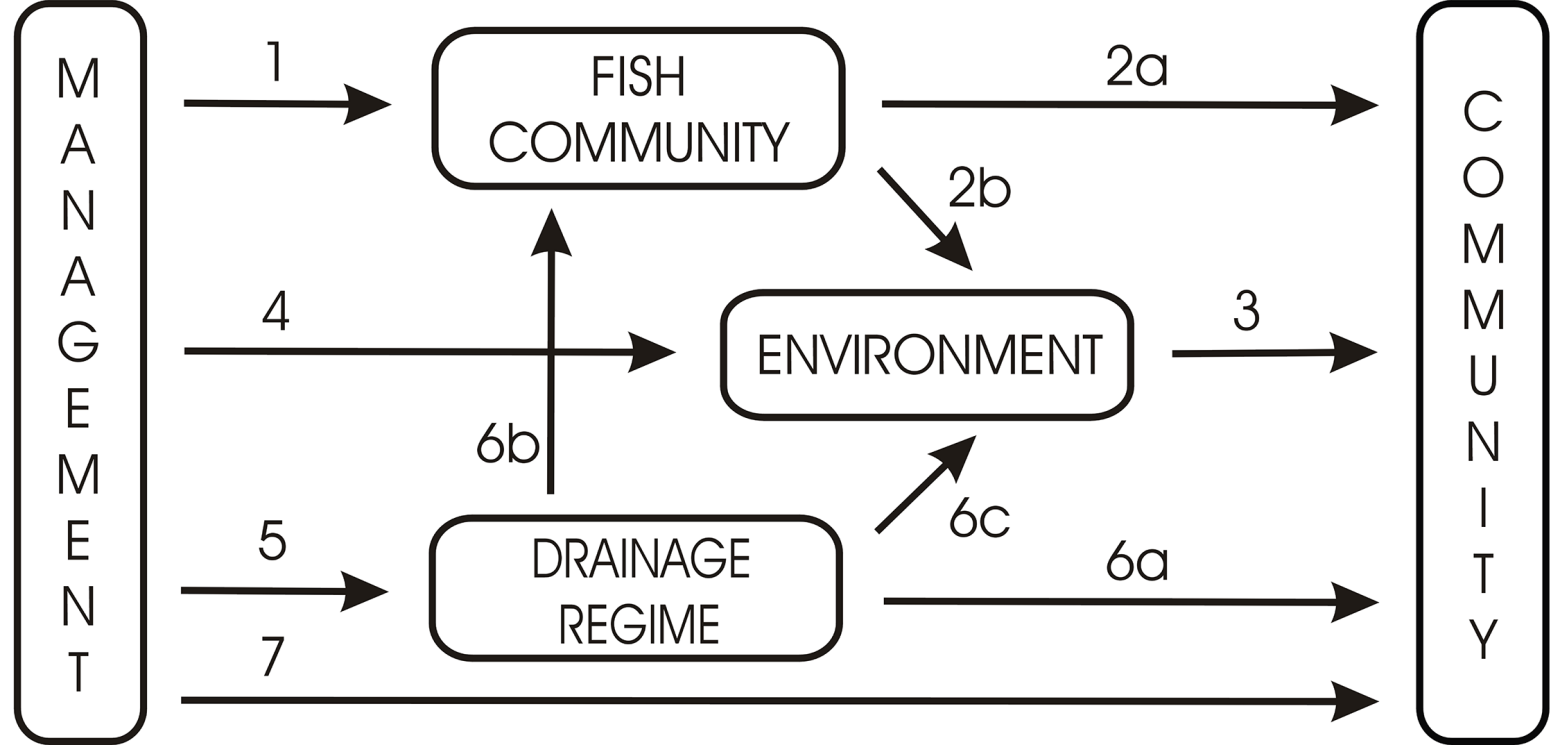
Conceptual model representing the potential direct and indirect ways through which pond management can affect aquatic communities. Management may determine fish community characteristics (1), which may affect other organism groups directly through the associated predation regime (2a) or indirectly via an alteration of the pond environment (2b+3). Management may also affect aquatic communities by altering local environmental pond conditions (4+3). In addition, management can also determine the pond drainage regime (5), which in its turn may have direct effects on aquatic communities (6a), or indirect effects through its impact on the fish community (6b+2a or 6b+2b+3) or the pond environment (6c+3). Management may also have unique effects independently of fish, drainage regime and the measured environmental variables (7).

In a complex of eutrophic man-made fish ponds in Belgium, Lemmens *et al*.[15] showed that ponds used for carp farming were characterized by low local diversity and a low contribution to regional biodiversity for a variety of aquatic organism groups. In contrast, a management directed at maximally preventing the establishment of fish populations by annual periodic winter drainage yielded high levels of local diversity with multiple rare and endangered species, and also contributed substantially to regional diversity. Current conservation management in this pond complex largely involves the mimicking of traditional fish farming activities, mainly through a combination of fish stock management and temporal drainage (Fig 1). Fish stock management can greatly influence the characteristics of fish communities and affect other organism groups directly through predation [17–20] or indirectly through their effect on the pond environment (e.g. sediment resuspension, nutrient cycling, physical disturbance) [21–23]. Drainage regime can have a strong direct impact on multiple organism groups [24–27], including fish, but may also have important indirect effects by altering pond environmental conditions [28–30]. In addition, management can affect aquatic biota via other changes in the pond environment through measures such as reductions of nutrient loading or sediment removal. Finally, pond management may affect pond biota directly, for example seed or resting egg banks that are removed together with sediments (Fig 1).

The present study aims at disentangling the relative importance of the direct and indirect ways through which conservation measures may affect the community composition of aquatic organisms in eutrophic, interconnected ponds. For this purpose, we re-analyzed part of the dataset of Lemmens *et al*. (2013) [15] using data on 28 ponds that equally represent four major types of conservation management differing in the intensity of fish stock management (no stocking of fish, stocking with fish fry, stocking of low density of fish) and the frequency of periodic pond drainage (annual, occasional, almost never). Although the present study uses largely the same data as Lemmens *et al*., (2013), its focus and approach are substantially different. First, here we focus on the effects of management on community composition rather than diversity. Second, we take a more mechanistic approach by investigating how pond management may affect aquatic communities through different alternative pathways as outlined in the conceptual model of Fig 1. Central questions are: (1) What is the relative importance of individual management measures (e.g. fish stock and drainage regime management), (2) To what extent do these factors act separately or in conjunction, via direct or via indirect pathways? (3) How do organism groups differ in their response?

## Methods

### Ethics statement

We sampled in accordance to the European directive 2010/63/EU and had explicit permission of respective owners (fish farmers and the Agency for Nature and Forests) to enter private property. Approval by an Institutional Animal Care and Use Committee or equivalent animal ethics committee is not required in Belgium for field sampling. No additional permissions were required for this study.

### Study area, pond selection and data collection

This study was performed in “Vijvergebied Midden-Limburg”, which is situated in the North-eastern part of Belgium (50°N, 5°E and surroundings) and is part of "De Wijers" area (see also [15]). The region is recognized for its large number of man-made shallow ponds (n > 1000) and is well known for being a biological hotspot of aquatic biodiversity at the regional scale. Most ponds are indirectly connected to the River Demer basin via two small streamlets and are interconnected by a complex network of rivulets. The conservation values in the region largely result from traditional extensive fish farming. This practice has increasingly been replaced by more intensive farming practices, which result in ecological degradation of the ponds. Fish farming is still an important local practice, but the majority of the ponds are currently designated as Natura 2000 sites. They are protected by the Birds directive (79/409/EEC) and the Habitats directive (92/43/ECC), and are solely managed for purposes of biodiversity conservation. Current conservation management is done by the Agency of Nature and Forests and largely aims to mimic traditional extensive fish farming practices by frequent periodic pond drainage and the stocking of low densities of fish in some ponds. Stocking of fish is mainly done for the conservation of Eurasian Bittern (*Botaurus stellaris)* and Common Little Bittern (*Ixobrychus minutus*). This type of interconnected ponds is representative to other fish pond complexes in European countries, such as France [31, 32], Germany [33], Poland [34], Hungary [35] and the Czech Republic [10].

We selected four types of biodiversity conservation management differing in the frequency of pond drainage and intensity of fish stock management (Table 1). Individual ponds frequently shift between management types. We randomly selected seven ponds from each of the four management types (n = 28) (S1 Fig). These ponds were surveyed either in 2006 or 2007 (2006, n = 15; 2007, n = 13) due to logistical constraints to sample all ponds in a time period short enough to avoid unwanted seasonal variation in the data. None of the ponds were sampled in both years. To avoid biases introduced by interannual variability, we made sure to equally represent all management types in both years (2006: No fish [NF], n = 3; Farming of Young of Year Fish [YF], n = 4; No Management [NM], n = 4; Low Intensity Fish Farming [LI], n = 4; 2007: No Fish, n = 4; Farming of Young of Year Fish, n = 3; No Management, n = 3; Low Intensity management, n = 3). We randomly selected the ponds to be sampled in 2006 and 2007. During each year, ponds of different management types were sampled in a random order.

**Table 1:** Description of the pond management types in relation to fish stock management and frequency of periodic pond drainage. These management types are currently applied in the region for purposes of biodiversity conservation.

Management Type	Purpose	Fish stock management	Frequency of drainage
No Fish (NF)	To create fishless ponds (mainly for amphibian conservation)	No stocking of fish. Nets of 2 mm mesh are placed on the inlets to avoid immigration of fish	Ponds are drained annually in autumn and refilled in early spring
Farming of Young of Year Fish (YF)	To maintain extensive fish farming practices that historically resulted in high conservation values	Stocking with fish fry (Ide [*Leuciscus idus]* and Common carp [*Cyprinus carpio]*) in late spring. Nets of 2 mm mesh are placed on the inlets to minimize immigration of other fish. Fish is harvested in autumn	Ponds are annually drained in autumn and are incrementally refilled in spring in order to allow development of lush emergent vegetation for YF
No Management (NM)	No specific purpose	No fish stock management. Fish can freely move in and out the ponds via rivulets	Last drainage more than ten years ago
Low Intensity Management (LI)	To maintain extensive fish farming practices that historically resulted in high conservation values	Two or three years prior to this study, ponds were drained, refilled and initially stocked with adult rudd (*Rutilus rutilus*), tench (*Tinca tinca*) and pike (*Esox lucius*) (total 40 kg ha^-1^). Fish can freely move in and out of the ponds	Ponds are occasional drained (approximately every five years, but irregularly spaced in time)

Pond surface, maximum pond depth and the thickness of the sediment layer were determined once during summer. We measured daytime oxygen and water temperature in spring and summer. Water samples from May and July were analyzed for water transparency, the concentration of chlorophyll *a*, nutrients (total nitrogen and phosphorus) and suspended solids. Fish, zooplankton and phytoplankton were sampled once in July, while samples from macro-invertebrate communities were collected twice a year (May and July) to incorporate seasonal variation in community composition. We visually estimated the percentages of pond area covered by submerged, floating and emergent vegetation during August. At that moment, we also inventoried the abundances of the different plant species in each pond. Pond surfaces were calculated using the GIS software package ArcView GIS 3.2a (ESRI, Inc.). Maximum pond depths were measured with a graduated stick at the deepest point of each pond. We estimated the thickness of the silt layer from the profile of sediment cores taken at 2 random chosen spots in the deeper part of the ponds. Standard electrodes (WTW multiline F meter, Geotech) were used to measure water temperature, pH and daytime oxygen concentration at one location in the open water at a depth of 20 cm in each pond. Water transparency was determined using a Snell tube [36]. A tube-sampler (length 1.2 m; diameter 75 mm) was used to take depth-integrated water samples in the pelagic zone at five locations in each pond in spring and summer. These water samples were pooled and subsamples were taken for further analysis of suspended solids, chlorophyll *a* and nutrient (nitrogen and phosphorus) concentrations. The concentration of suspended solids in the water column was determined gravimetrically by filtering pond water through GF/F filters (Whatmann). We measured chlorophyll *a* concentrations spectrophotometrically following Ritchie [37] after methanol extraction [38]. Total concentrations of nitrogen (TN) and phosphorus (TP) were determined after alkaline persulfate digestion [39] on a Technicon Auto Analyzer II (Technicon, Tarrytown, New York, USA).

Fish community characteristics were determined by placing multiple (n = 3–5, dependent on pond surface area) double fyke nets (length 7.7 m, mesh size 8mm) in each pond for 24 hours. All specimens were identified to species level, measured (fork length) and weighed in the field. The total fish biomass and the total biomass of each species per pond was expressed as catch per unit effort (CPUE: kg per fyke net). We sampled zooplankton and phytoplankton communities quantitatively by collecting depth-integrated water samples at five randomly chosen locations in the littoral and pelagic zone of each pond using a tube-sampler. A beaker was used in very shallow habitats. Zooplankton communities were sampled by filtering 40 L of the pooled water sample through a conical plankton net (mesh size, 64 μm). We collected 250 mL from the pooled sample to characterize the phytoplankton community. Cladocerans were identified to species level [40] and counted. *Daphnia galeata* and *D*. *longispina* were considered as one taxon. Copepods were divided in two main groups (Calanoida and Cyclopoida) and counted. Phytoplankton was identified to genus level [41]. Aquatic macro-invertebrates were sampled in the littoral zone of each pond by sweeping with a D-shaped net (23 cm x 23 cm, 500 μm mesh size) during 10 minutes in total [42]. The sampling time for different mesohabitats (submerged, floating and emergent vegetation) was in proportion to their relative abundance. Samples from different mesohabitats were pooled in the field. Ephemeropterans, hemipterans and molluscs were identified to species level. Identification of dipterans was done to family level. Lepidoptera, Hirudinea, Assellidae and Gammaridae were only counted. The plant species abundance was inventoried using the Tansley scale (rare, occasional, frequent, abundant, dominant) [43], which was converted to an ordinal scale ranging from 1 to 5 respectively prior to statistical analysis. Ordinal scaling was also used to define the frequency of pond drainage (0 = last drainage > 10 years ago, 1 = occasional drainage, 2 = annual drainage). We used average values for local environmental pond variables that were measured twice a year and took the sum of macro-invertebrate abundances from May and July for all analyses to take into account seasonality in community composition. Abundance data from the single sampling campaign were used for the analysis of phytoplankton, zooplankton and aquatic plant communities.

### Data analysis

We applied variation partitioning analyses to explore the relative importance of direct and indirect effects of pond management on the community composition of each of the investigated organism groups. Variation partitioning analysis allows partitioning the total amount of variation explained by a statistical model into unique and shared contributions of sets of predictor variables [44, 45]. We carried out separate analyses for phytoplankton, submerged plants, emergent vegetation, zooplankton and macro-invertebrates. With regard to the latter, we first applied an overall analysis including all investigated taxa at the family level (except for Hirudinae and Lepidoptera, which were included at the level of subclass and order, respectively) (further referred to as the “macro-invertebrates”). In addition, we exploited the higher level of taxonomic detail available for Mollusca and Hemiptera and analyzed the community compositional variation of these groups also separately at the species level. These analyses provide additional information since an analysis at the family level only may obscure group-specific response patterns resulting from the large differences in life history characteristics, feeding ecology, dispersal mode and physiology that exist among invertebrate groups.

Using redundancy analyses (RDA), we first evaluated the effect of pond management type on the community composition of each of the target organism groups, as well as on fish community composition and on the entire set of measured local environmental pond variables. Second, we separately evaluated the effect of fish community, environmental variables and drainage frequency on community composition of each organism group. We did not specifically test for the effect of management on drainage frequency, since drainage is an intrinsic feature of the investigated pond management types. The associations between pond management types, the local pond environment, fish communities and community composition of target organism groups were also visually investigated using ordination plots produced by Principal Component Analyses [45].

Third, for each target organism group separately, we partitioned the amount of explained community variation between each significant set of explanatory variables (fish community composition, environmental variables and the frequency of drainage) irrespective of pond management type. The aim of these analyses was to reveal the relative importance of these three main drivers behind community variation, and their potential unique and shared effects. The interpretation of a significant unique contribution of a variable set is straightforward and indicates a direct effect, independent of the other variable sets in the model. Shared contributions may result from indirect effects with one factor having an effect through its impact on the other explanatory factor, but can also arise as the result of a common response to a same latent factor. However, the interpretation is often straightforward when it concerns a known causal relationship. For example, when explained variation is shared between drainage frequency and fish composition, this may indicate effects of drainage on target communities through its impact on the fish community, whereas the opposite is impossible. A similar reasoning can be made for a shared component of drainage frequency and the local environmental variables.

Fourth, we repeated the variation partitioning analyses, also including management type as an explanatory variable category. For the interpretation of the importance of management, we mainly focused on three components of explained variation: (1) the fraction of variation in community composition that can uniquely be attributed to pond management type. This component may reflect the impact of direct management effects independently of fish, drainage regime and the measured environmental variables, although mediation by other unknown environmental factors cannot be excluded; (2) the variation that can uniquely be attributed to the ensemble of fish, drainage and environmental variables, which quantifies what can be explained by these explanatory variables independent of pond management; and (3) variation that can be explained both by management type and the ensemble of fish, drainage and environment, indicating the total amount of variation that is potentially caused by the indirect effects of pond management type through fish, drainage or the measured environmental variables.

In all analyses that included fish community composition in the set of explanatory variables, fish compositional variation was represented by the sample scores on the first two axes of a principal component analysis on fish biomass composition (PCA) [46]. The effect of pond environment on each target organism groups was assessed based on the entire set of measured environmental variables. In the variation partitioning analyses, however, we only retained significant environmental variables identified using forward selection based on the adjusted R^2^ double stopping criterion [47]. The percentage of pond surface covered with submerged vegetation and with reed and emergent vegetation were excluded from the analyses of submerged and emergent vegetation community composition, respectively, to avoid artifacts caused by collinearity. We refer to S1 Table for an overview of the variables included in each step of the statistical analysis. S2 Table provides the results from Spearman correlations between the explanatory variables that were included in the RDA analyses for the different organism groups.

Fish biomass data and all environmental pond variables, except pH, were logarithmically transformed prior to statistical analysis. Taxon abundances of the different taxonomic groups were Hellinger-transformed [48]. The significance of all RDA models was assessed with Monte-Carlo permutations (n = 999) restricted within sampling year (2006 and 2007)[45, 49]. Adjusted R^2^ values were calculated on the residuals after partialling out the effect of sampling year. All statistical analyses were carried out in R version 3.0.1 (R Development Core Team 2013) using the rda and varpart functions of the vegan library [44, 50].

## Results

### Effect of management type on community composition of target groups

**Fig 2:**
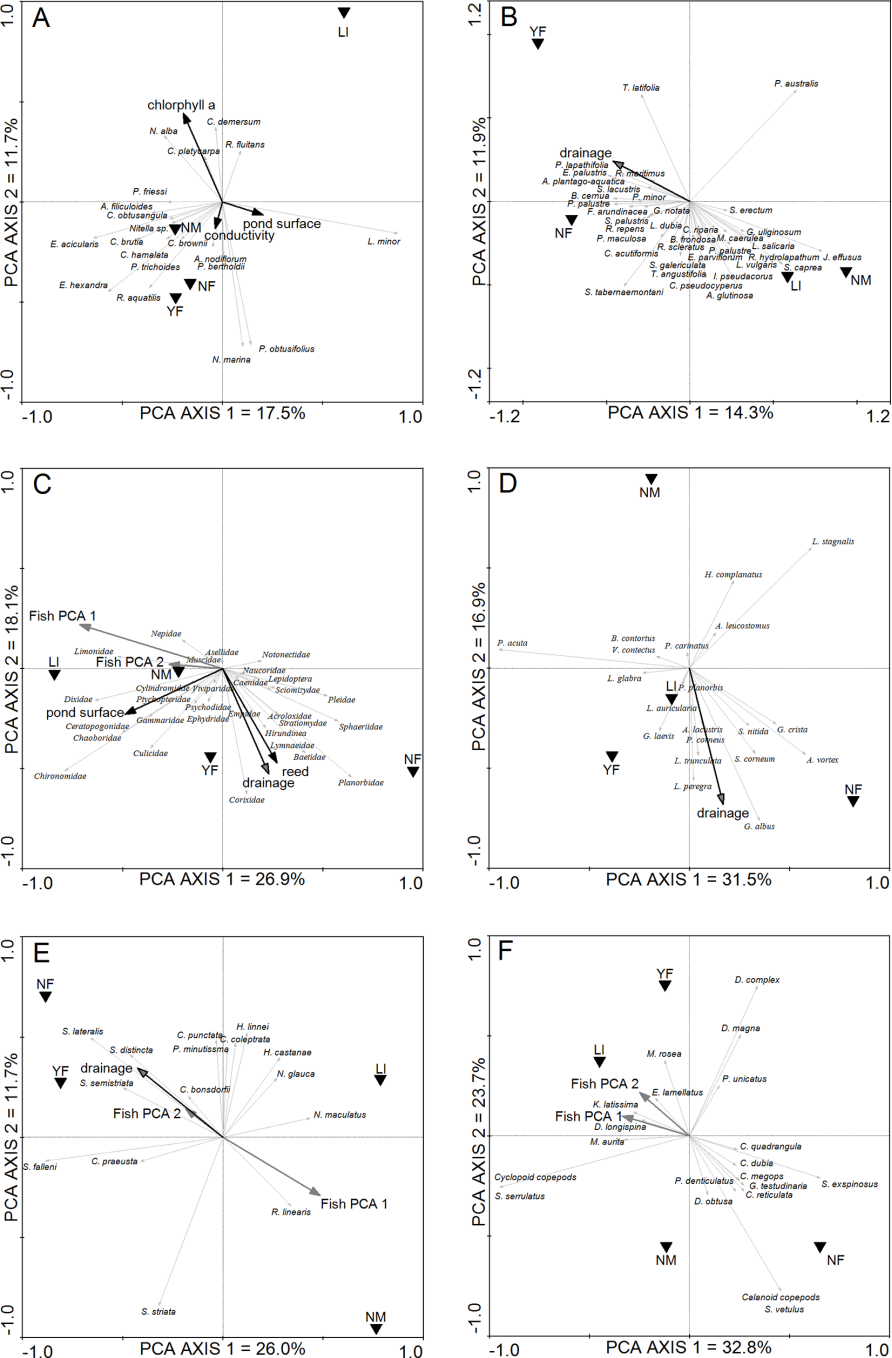
Ordination plot of PCA analysis on the community composition the investigated organism groups. (A) submerged and floating vegetation, (B) emergent vegetation, (C) macro-invertebrates, (D) mollusks, (E) hemipterans, (F) zooplankton. Only significant explanatory variables are visualized for each taxonomic group. Black triangles represent the centroids of the pond management types, grey arrows represent the first two site scores vectors of the PCA on fish community data, black arrows represent significant environmental variables and black arrows with grey fill represent frequency of pond drainage. Pond management type, site scores vectors of fish community, significant environmental variables and frequency of pond drainage were plotted as supplementary variables so did not influence the ordination. For clarity, only the taxa that occurred in minimum 15% of the samples are shown for submerged, emergent plants and zooplankton. The ordination plot of phytoplankton community composition is not shown since none of the explanatory variable sets had a significant effect. See S3 Table for full species names.

Pond management type significantly explained variation in community composition of all target organism groups except phytoplankton (Table 2). Most macrophytes were negatively associated with LI ponds, whereas a considerable number of species showed a positive association with NF and YF ponds (Fig 2). Communities of emergent plant species in NF and YF ponds were characterized by several disturbance resistant, pioneering species, whereas late successional species tended to be more important in LI and NM ponds. Macro-invertebrate community composition differentiated the NF management from the LI management type. Many mollusk species showed a positive association with the NF management and were negatively associated with the NM management. The majority of zooplankton species showed a positive association with the NF management (Fig 2). We refer to S1 File for a more detailed analysis and description of community responses to management type.

**Table 2:** Results of RDA analyses. RDA analyses separately testing for effect of pond management type (MAN), fish community composition (FISH), local environment variables (ENV) and frequency of pond drainage (DRAIN) on the community composition of each of the studied target organism groups.

		df	F	R^2^ ^a^	p ^b^	sign. env. var. ^c^
Phytoplankton	MAN	3	1.09	1%	ns	
	FISH	2	1	0%	ns	
	ENV	13	1.14	7%	ns	
	DRAIN	1	1.37	1%	ns	
Submerged and	MAN	3	1.5	5%	*	
floating vegetation	FISH	2	1.38	3%	ns	
	ENV	12	1.34	14%	*	surface area, cond, chla
	DRAIN	1	1.37	1%	ns	
Emergent vegetation	MAN	3	1.25	3%	*	
	FISH	2	1.32	2%	ns	
	ENV	12	1.13	6%	ns	
	DRAIN	1	1.48	2%	*	
Hemipterans	MAN	3	1.44	6%	*	
	FISH	2	1.7	6%	*	
	ENV	14	1.03	2%	ns	
	DRAIN	1	2.17	5%	*	
Mollusks	MAN	3	2.2	14%	**	
	FISH	2	1.16	1%	ns	
	ENV	14	1.04	2%	ns	
	DRAIN	1	2.65	7%	**	
Macro-invertebrates	MAN	3	2.23	14%	**	
(family level)	FISH	2	2.96	15%	**	
	ENV	14	1.32	17%	**	surface area, reed
	DRAIN	1	2.8	8%	**	
Zooplankton	MAN	3	1.84	8%	*	
	FISH	2	1.4	3%	*	
	ENV	14	0.83	0%	ns	
	DRAIN	1	1.06	0%	ns	

^a^ The percentage of explained variation (i.e. marginal effects).

^b^ The significance level

'*' p <0.05

'**' p <0.01; 'ns' not significant.

^c^ Significant environmental variables that were selected by the forward selection procedure.

### Effect of management type on fish communities and the pond environment

**Fig 3:**
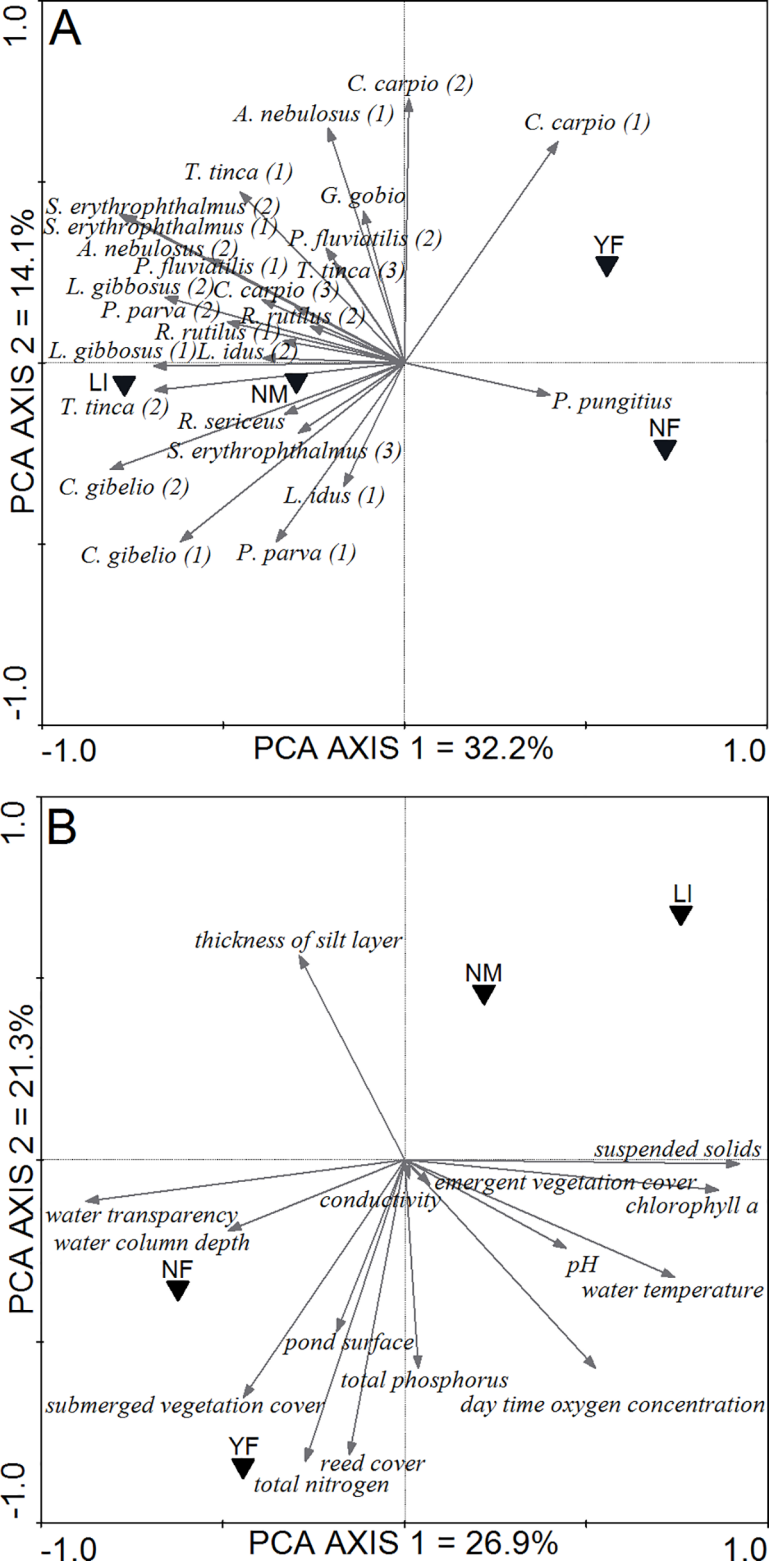
Ordination plots of Principal Component Analyses (PCA). (A) PCA plot on fish biomass composition and (B) a standardized PCA plot on pond environment variables. Black triangles represent the centroids of the pond management types and were plotted as supplementary variables, in order not to influence the ordination. Numbers behind the fish species names indicate different size classes (1: < 10 cm, 2: >10 cm and 3: >20 cm; except for *L*. *gibbosus*, *P*. *parva* where 1: < 7cm and 2: > 7cm; for *G*. *gobio*, *P*. *pungitius* and *R*. *sericeus* no differentiation in size classes was made). *A*. *nebusolus* = *Ameiurus nebulosus*, *C*. *gibelio* = *Carassius gibelio*, *C*. *carpio* = *Cyprinus carpio*, *G*. *gobio* = *Gobio gobio*, *L*. *gibbosus* = *Lepomis gibbosus*, *L*. *idus* = *Leuciscus idus*, *P*. *fluviatilis* = *Perca fluviatilis*, *P*. *parva* = *Pseudorasbora parva*, *P*. *pungitius* = *Pungitius pungitius*, *R*. *sericeus* = *Rhodeus sericeus*, *R*. *rutilus* = *Rutilus rutilus*, *S*. *erythrophthalmus* = *Scardinius erythrophthalmus*, *T*. *tinca* = *Tinca tinca*.

RDA analyses revealed that management type significantly explained variability in fish community composition (F = 2.126, R^2^ = 11.9%, p = 0.005). Management type also had profound effects on local environmental conditions (F = 4.983, R^2^ = 31.49%, p = 0.001). Sample scores on the first axis of the fish community PCA (eigenvalue = 0.322) were strongly correlated with total fish community biomass (Pearson correlation, r = 0.67, p < 0.001) and clearly differentiated NF and YF ponds from LI and NM ponds (Fig 3A). The second PCA axis comprised considerably less variation (eigenvalue = 0.141) and differentiated YF ponds from NF ponds. Most size classes of most fish species showed a clear positive association with the LI and NM management, except small size classes of common carp, which tended to be more abundant in YF ponds. Nine-spined stickleback (*Pungitius* p*ungitius*) was the only species with highest abundances in NF ponds. These ponds were thus not fish free but contained low densities of topmouth gudgeon (*Pseudorasbora parva*), pumpkinseed sunfish (*Lepomis gibbosus*), gibel carp (*Carassius gibelio*) and nine-spined stickleback (Fig 3A).

The first and second axis of the standardized PCA from the environmental variables set jointly represented 48.2% of the variation in pond environment and strongly differentiated NF and YF ponds from NM and LI ponds (Fig 3B). Water transparency was positively associated with NF ponds and to a lesser extent also with YF ponds. Conversely, high concentrations of suspended solids and chl *a* were characteristic for LI ponds. The percentage of pond surface covered with submerged vegetation and reed was positively associated with YF and NF ponds. Nutrient concentrations (total nitrogen and total phosphorus) tended to be higher in YF ponds compared to LI and NM ponds. Conductivity and the percentage of pond surface covered with emergent vegetation did not significantly differ between management types (Fig 3B).

### Effects of fish community, pond environment and drainage frequency on the community composition of target groups

**Fig 4:**
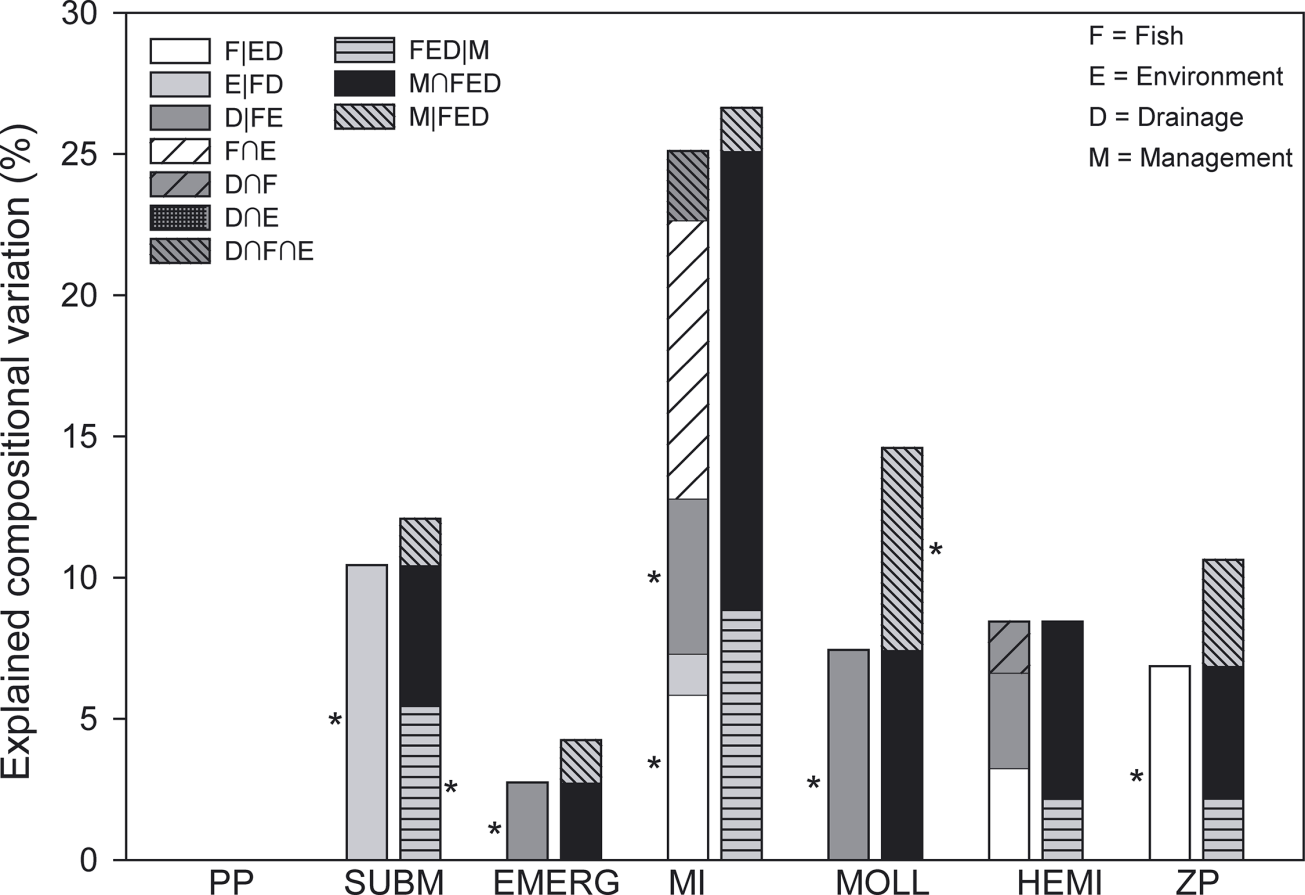
Stacked bars showing the results of variation partitioning analyses on community composition of phytoplankton (PP), submerged and floating vegetation (SUBM), emergent vegetation (EMERG), aquatic macro-invertebrates (MI), mollusks (MOLL), Hemipterans (HEMI), and zooplankton (ZP). The left-hand bars show compositional variation uniquely explained by fish (F|ED), pond environmental variables (E|FD) and pond drainage (D|FE), as well as the fraction of variation that is commonly explained by fish and environment (F∩E), drainage and fish (D∩F), drainage and environment (D∩E) and by drainage, fish and environment (D∩F∩E). Right-hand bars show the proportion of variation that is commonly explained by fish, pond environment and drainage independently from management (FED|M), the proportion of variation commonly explained by pond management, fish, pond environment and drainage (M∩FED), and the proportion of variation that is uniquely explained by pond management (M|FED). Proportions of variation that are significantly explained are indicated with * (P<0.05). Note that the significance of the fractions F∩E, D∩F, D∩E, D∩F∩E and M∩FED cannot be tested.

The degree to which the composition of communities was affected by fish, pond environment and drainage frequency differed considerably among the studied organism groups (Table 2). Variation partitioning analyses revealed clear and unique associations of fish community characteristics with the community composition of macro-invertebrates and zooplankton, independently of the pond environment or the drainage regime (R^2^_adj._ of conditional effects 5.95% and 6.86%, respectively, p < 0.05) (Fig 4). In addition, fish community also explained a proportion of macro-invertebrate compositional variation in concert with pond environmental variables (R^2^_adj._ = 9.85%). Drainage frequency had unique effects on communities of emergent macrophytes, the whole group of macro-invertebrates and mollusks (R^2^_adj._ ranging from 2.74% to 7.44%, p < 0.05), and also explained hemipteran community composition in common with fish community (R^2^_adj._ = 3.97%; Fig 4). Although local environmental conditions in the pond were the only variables explaining variation in submerged macrophyte community composition (R^2^_adj._ = 10.44%), their unique contribution to the other organism groups was relatively minor or non-existent. None of the categories of explanatory variables were able to explain variation among phytoplankton communities.

### The relative importance of unique and confounded effects of pond management type on the community composition of target organism groups

Effects of fish community, local environmental conditions or the frequency of pond drainage were found to be highly confounded with pond management type. Effects of any of these three variable categories became insignificant when pond management type was accounted for in a variation partitioning analysis, except for submerged macrophytes (Fig 4). A more detailed presentation of the results of these variation partitioning analyses involving pond management type is provided in S2 Fig. Pond management type affected target organism groups almost entirely indirectly through its impact on fish community, pond environment or drainage frequency, because its effects became insignificant upon controlling for the other three explanatory variable categories (Fig 4 and S1 Fig). Mollusks formed the only exception to this.

The pathways through which pond management affected communities varied strongly between the different organism groups. Pond management type affected submerged macrophyte community composition mainly through its effects on the pond environment, whereas management-associated drainage frequency was important for emergent plant and mollusk communities. A relatively large amount of variation in macro-invertebrate community was explained through effects of management type on the fish communities and the pond environment. Additional amounts of macro-invertebrate compositional variation was explained by direct and indirect effects of management-associated drainage frequency. Management type affected hemipteran communities mainly through direct and indirect effects of drainage frequency, while management type affected zooplankton communities mainly indirectly through its effect on fish community characteristics. We refer to S2 File for a detailed description of the pathways by which management affected each target organism group.

## Discussion

The present study provides evidence for effects of pond management on the community composition of a diverse array of aquatic organism groups in fish ponds. Our analyses indicate that pond management type affected the investigated communities mainly through its associated drainage and fish stocking regimes. Independent variation in fish communities or local environmental variables provided no significant additional explanatory power to pond management type and its associated drainage regimes (with the exception of the response of the communities of macrophytes). Furthermore, the effects of the fish community and drainage frequency tended to be largely independent of each other. Whether management type affected communities directly or through its impact on fish communities, drainage regime or local environmental conditions, however, varied considerably between organism groups. Overall, the abundances of the majority of investigated biota were negatively associated with fish density, whereas drainage frequency had positive effects on multiple emergent plant species and several taxa of aquatic macro-invertebrates.

The relatively strong association between fish community characteristics and the community composition of macro-invertebrates, hemipterans and zooplankton, independently of pond environment and drainage frequency, suggests fish to be an important driver through which pond management affects these organism groups. Indeed, fish are known to be efficient, often highly selective visual predators with the capacity to shape the species composition and size distributions of their prey communities [12, 18, 51]. Although direct predation has been suggested as the major mechanism by which fish determine the characteristics of invertebrate communities in ponds [52], fish community characteristics may also have considerable indirect effects on habitat selection and colonization of the active dispersers [53, 54].

Our results also suggest that fish stock management has considerable indirect effects on the whole group of macro-invertebrates by altering the pond environment. A proportion of the explained macro-invertebrate community variation was shared between fish and the pond environment, more specifically pond surface area and the degree to which the shoreline was covered by reed. Reed on the shoreline showed a strong negative correlation with the first fish community PCA axis (r = -0.55, p = 0.005), which suggests that fish affected the growth of reed negatively, e.g. by disturbing sediments [22] or by consuming or damaging young shoots [55]. It is very likely that this has impacted the composition of the macro-invertebrate communities. The lack of significant effects of other environmental variables on the community composition of macro-invertebrates is in line with other studies. Indeed, previous studies show that the spatial distribution and abundance of many invertebrates is primarily driven by the presence of fish, rather than by abiotic conditions [56–58]. Alternatively, the absence of any effect of environmental variables may also have resulted from the relatively low taxonomic resolution (family level) in this analysis.

Drainage was the only factor through which pond management affected the community composition of emergent vegetation and mollusks, apart from some unique effects of management type in the latter group. Periodic pond drainage also affected the whole group of macro-invertebrates and hemipteran community composition. High frequency of winter drainage favored typical disturbance resistant pioneering emergent plant species, whereas late-successional species were more prominent in ponds that were rarely or never drained. This finding is supported by other studies, which have shown that periodic drainage alters aquatic vegetation assemblages [59, 60] by reducing vegetation succession rate and promoting pioneering vegetation [11].

Multiple mollusk species and several families of macro-invertebrates were positively associated with high frequency of pond drainage, independently of the management-dependent intrinsic association between drainage and fish community composition. Periodic drainage is often considered a harsh environmental filter that may cause many invertebrate species to go locally extinct [24, 61]. Frequent drought events may, however, also have profound positive effects on multiple taxa of macro-invertebrates by (1) weakening competitive exclusion which favors co-existence of species [62, 63], or (2) by preventing the population build-up of large invertebrate predators, such as odonate and coleopteran larvae, which may otherwise reduce the abundance of other invertebrates [64]. Organisms with drought resistant propagules may survive as dormant stages and can rapidly establish new populations after ponds refill, whereas other organisms depend entirely on recolonization from the regional species pool. Our results indicate that recolonization has been rapid for macro-invertebrates and mollusks, a process that may have been strongly facilitated by the high degree of connectivity between ponds via rivulets and overflows [65] and the high density of neighboring ponds that were not simultaneously drained.

Pond management type affected submerged macrophyte community composition entirely through the pond environment. In addition, pond environment also explained a relatively large proportion of compositional variation in submerged macrophytes independently of management. Effects of management type were largely mediated through variation in concentrations of phytoplankton chlorophyll *a*, whereas pond surface accounted for the unique effect of the pond environment. High concentrations of chlorophyll *a* reduce light penetration in the water column, which ultimately leads to competitive exclusion [66] and selection towards shade-tolerant submerged macrophyte species [31]. In the present study, shade-tolerant species such as *Nymphaea alba* and *Ceratophyllum demersum* tended to be more important in ponds with high biomass of phytoplankton as measured by the concentration of chlorophyll a. The majority of submerged plant species showed a negative association with pond surface, which might be attributed to the increasing exposure to wind and wave disturbance with increasing pond size [67]. In contrast to previous investigations [11, 59, 60], we found no evidence for effects of drainage on the community composition of submerged vegetation. Usio *et al*. [68] obtained similar results and suggested that winter drainage may only have a minor impact on submerged macrophyte communities in highly connected pond systems since many desiccation sensitive species rapidly recolonize as seeds from the seed bank or turions that are efficient in dispersing from neighboring, non-drained ponds [69].

In conclusion, our study shows that management affects a variety of aquatic assemblages in former fish farming ponds. Furthermore, our results indicate that the direct and indirect effects through which human management alters the community composition of aquatic organisms varies strongly between taxonomic groups. With the exception of submerged macrophytes, the impact of fish community characteristics and pond drainage regime on the community composition of target organism groups seemed to be more important than other characteristics of the pond environment.

Lemmens *et al*. [15] demonstrated a strong relation between pond management type and the diversity of multiple organism groups, and found that low fish density and periodic pond drainage promotes the diversity of multiple organism groups. The present study uses largely the same dataset but helps with identifying the major mechanisms through which different conservation measures affect the community composition of aquatic biota. Regular winter drainage seems to reset the successional stage by reducing competitive exclusion and promoting the establishment of early pioneering emergent vegetation without major negative effects on the communities of other biota. Succession is a major issue in current pond conservation management [70, 71] since it may eventually result in the loss of valuable habitats due to terrestrialization, especially in eutrophicated systems. The process of succession can be rapid and causes important temporal variability in conservation value of individual ponds [70]. Since different successional stages are often characterized by distinct communities [71], management programs should aim at sustaining different stages of succession at the landscape scale. In addition, pond drainage often allows efficient fish stock management and only requires limited financial and human resources. We therefore advocate that pond drainage is an important element in the tool box of managers of eutrophic shallow and interconnected man-made ponds. However, before deciding to drain ponds, managers should always take into consideration the regional context. Temporal drought events inevitably result in the extirpation of local populations and successful recolonization of refilled ponds largely depends on the distance to, or hydrological connection with, source populations in the surrounding landscape. This is particularly the case for large passively dispersing organisms and vertebrates. Periodic drainage may therefore not be preferable in ponds that are isolated or contain a unique and vulnerable fauna or flora. Management is likely not needed nor desirable in natural pristine waterbodies.

## Supporting Information

S1 Fig *Overview of a part of "Vijvergebied Midden-Limburg" with* the selected ponds representing the different management types. Note that one NF-pond, situated approximately 2 kilometers east of the depicted ponds, is not drawn on the map.
(DOCX)

S2 Fig Venn diagrams presenting the unique and shared contribution of fish community characteristics (FISH), pond environment (ENV), frequency of pond drainage (DRAIN) and pond management type (MAN) on the community composition of the investigated organism groups. (A) phytoplankton, (B) submerged and floating vegetation, (C) emergent vegetation, (D) macro-invertebrates, (E) mollusks (F) hemipterans and (G) zooplankton. Percentages outside the diagrams represent the R^2^-adjusted of the marginal effects of each significant explanatory set of variables. Percentages within the diagrams represent the R^2^-adjusted of the conditional effects of each set of explanatory variables. Asterisks denote the significance level, '*' p <0.05; '**' p <0.01; 'ns' not significant. Diagrams of pond management and pond drainage are shown in bold black and black respectively to indicate that their effects are unidirectional, which means that variability in management and frequency of drainage are not determined by fish community or pond environment.
(DOCX)

S1 File The effect of pond management type, fish community, pond environment and frequency of drainage on the composition of the investigated organism groups. (DOCX)

S2 File A detailed description of the pathways through which pond management type affected the investigated organism groups. (DOCX)

S1 Table Detailed overview of the variables that were used in each statistical analysis. MAN = pond management type, FISH = fish community, ENV = local pond environment, DRAIN = frequency of pond drainage. PP = phytoplankton, SUBM = submerged and floating plants, EMERG = emergent plants, MOLL = mollusks, HEMI = hemipterans, MI = macro-invertebrates, ZP = zooplankton. Note that no variation partitioning analysis was done for phytoplankton since MAN, ENV, FISH nor DRAIN had an overall significant effect. Abundance data of organism groups were Hellinger transformed.
(DOCX)

S2 Table Results from Spearman correlations between significant explanatory variables included in the RDA analyses for the different organism groups. (DOCX)

S3 Table Overview of all taxa our dataset and their occurrence in each management type (NF = No Fish, YF = Young of the Year Fish, NM = No Management, LI = Low Intensity Management). Species with abbreviations are shown on the PCA ordination plots (Fig 2). Emergent plants were classified based on the CSR strategy. Classifications of the CSR strategy between parentheses were derived from knowledge of the authors. The Grime’s CSR classification of plants essentially classifies plant species according to the three trade-off strategies for survival: competitor (C), stress tolerant (S) and ruderal (R). These strategies each thrive best in a combination of either high or low intensity of stress and disturbance.
(DOCX)
