# Supplementary material for: The Impact of Conservation Management on the Community Composition of Multiple Organism Groups in Eutrophic Interconnected Man-Made Ponds
Source: PLoS One. 2015 Sep 30;10(9):e0139371. doi: 10.1371/journal.pone.0139371 (PMC4589289; doi:10.1371/journal.pone.0139371)
Supplement: S1 Fig — Note that one NF-pond, situated approximately 2 kilometers east of the depicted ponds, is not drawn on the map. (DOCX) [file pone.0139371.s001.docx]

**
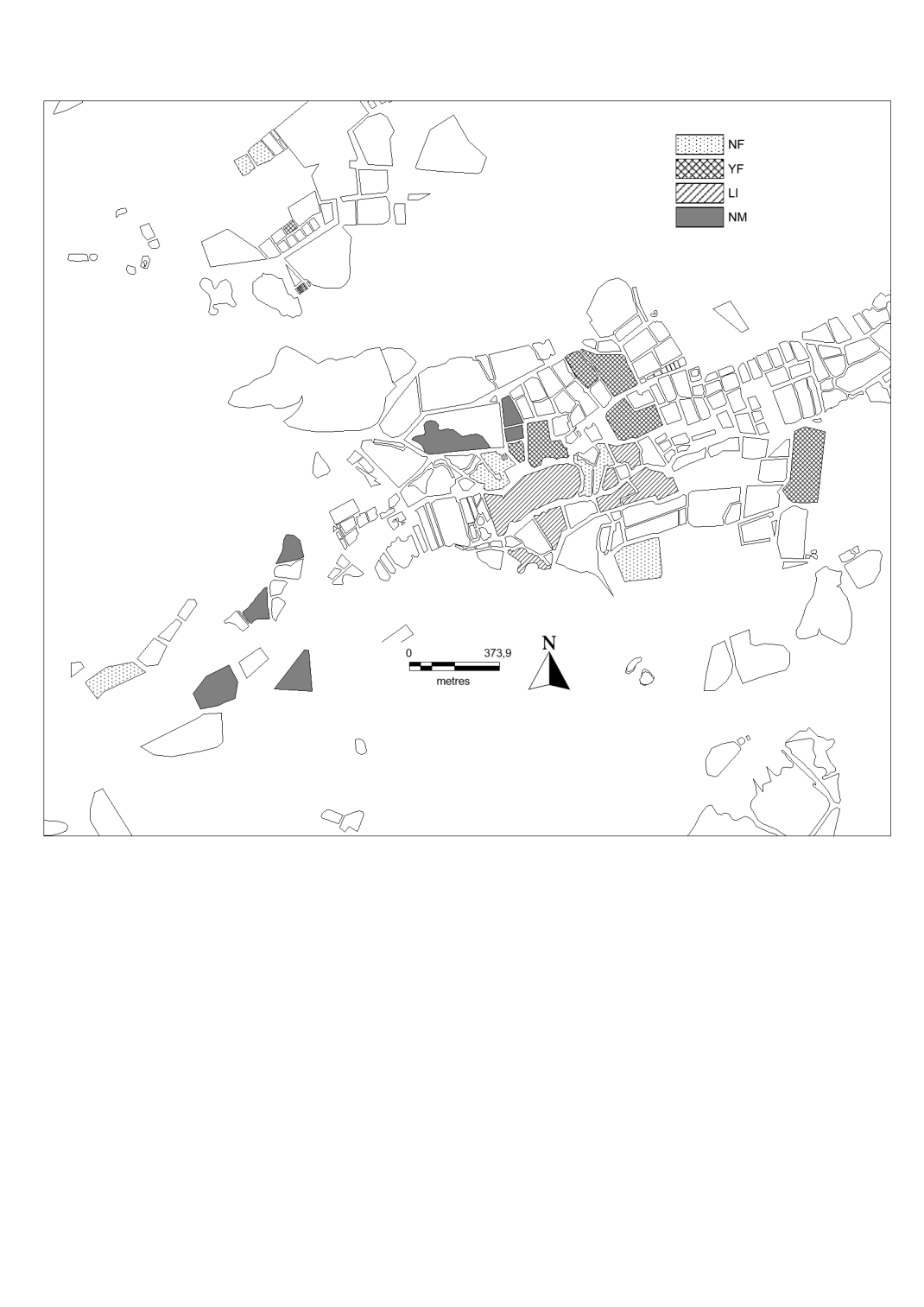
**

**S1 Fig. Overview of a part of "Vijvergebied Midden-Limburg" with the selected ponds representing the different management types.** Note that one NF-pond, situated approximately 2 kilometers east of the depicted ponds, is not drawn on the map.
