# Supplementary material for: The Impact of Conservation Management on the Community Composition of Multiple Organism Groups in Eutrophic Interconnected Man-Made Ponds
Source: PLoS One. 2015 Sep 30;10(9):e0139371. doi: 10.1371/journal.pone.0139371 (PMC4589289; doi:10.1371/journal.pone.0139371)
Supplement: S2 Fig — (A) phytoplankton, (B) submerged and floating vegetation, (C) emergent vegetation, (D) macro-invertebrates, (E) mollusks (F) hemipterans and (G) zooplankton. Percentages outside the diagrams represent the R2-adjusted of the marginal effects of each significant explanatory set of variables. Percentages within the diagrams represent the R2-adjusted of the conditional effects of each set of explanatory variables. Asterisks denote the significance level, '*' p <0.05; '**' p <0.01; 'ns' not significant. Diagrams of pond management and pond drainage are shown in bold black and black respectively to indicate that their effects are unidirectional, which means that variability in management and frequency of drainage are not determined by fish community or pond environment. (DOCX) [file pone.0139371.s002.docx]

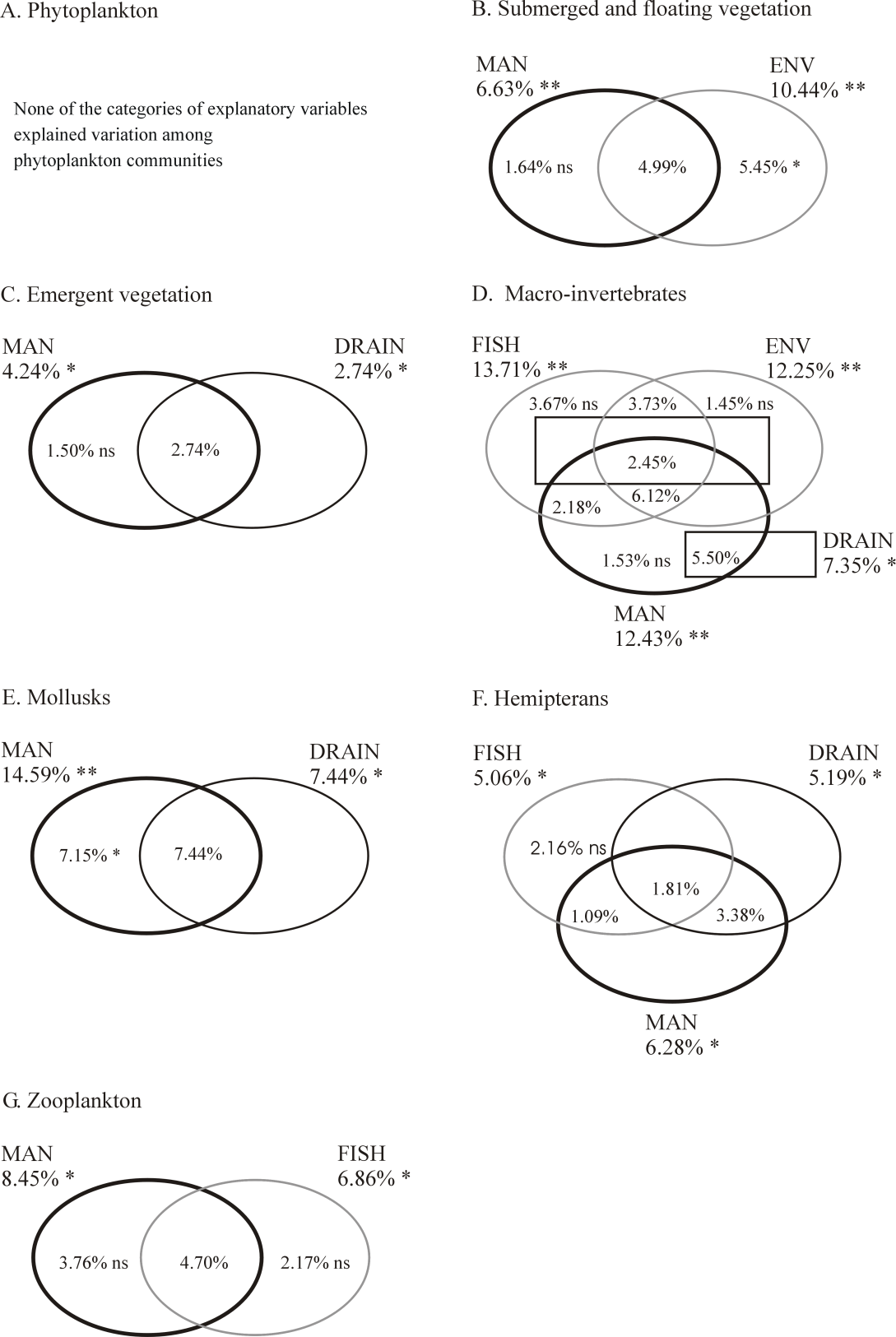


**S2 Fig. Venn diagrams presenting the unique and shared contribution of fish community characteristics (FISH), pond environment (ENV), frequency of pond drainage (DRAIN) and pond management type (MAN) on the community composition of the investigated organism groups.** (A) phytoplankton, (B) submerged and floating vegetation, (C) emergent vegetation, (D) macro-invertebrates, (E) mollusks (F) hemipterans and (G) zooplankton. Percentages outside the diagrams represent the R^2^-adjusted of the marginal effects of each significant explanatory set of variables. Percentages within the diagrams represent the R^2^-adjusted of the conditional effects of each set of explanatory variables. Asterisks denote the significance level, '*' p <0.05; '**' p <0.01; 'ns' not significant. Diagrams of pond management and pond drainage are shown in bold black and black respectively to indicate that their effects are unidirectional, which means that variability in management and frequency of drainage are not determined by fish community or pond environment.
