# Supplementary material for: The Impact of Conservation Management on the Community Composition of Multiple Organism Groups in Eutrophic Interconnected Man-Made Ponds
Source: PLoS One. 2015 Sep 30;10(9):e0139371. doi: 10.1371/journal.pone.0139371 (PMC4589289; doi:10.1371/journal.pone.0139371)
Supplement: S1 File — (DOCX) [file pone.0139371.s003.docx]

**S1 File. The effect of pond management type, fish community, pond environment and frequency of drainage on the composition of the investigated organism groups**

We found no effect of pond management type on the community composition of phytoplankton.

The first and second axes of the PCA ordination plot of submerged plant community jointly explained 29.3% of the compositional variation in the set of investigated ponds and differentiated the LI management from the NF, YF and NM management type (Fig. 2A in main text). Most macrophyte species were negatively associated with the LI management, whereas species typical for oligotrophic to mesotrophic conditions, such as *Potamogeton bertholdii*, *Potamogeton obtusifolius* and *Najas marina*, were positively associated with the NF and YF management. Forward selection of pond environmental variables indicated that variability in chl *a* concentration, conductivity and pond surface were major environmental drivers for compositional variation in submerged macrophyte community. Several species were positively associated with conductivity, and a very limited number of species, such as *Nymphaea alba*, *Ceratophyllum demersum* and *Riccia fluitans*, showed a positive association with phytoplankton chl *a* concentration. *Lemna minor* tended to be more abundant in larger ponds.

Emergent plant community composition in NF and YF ponds differentiated from those in NM and LI ponds (Fig. 2B). Disturbance tolerant species such as *Persicaria lapathifolia*, *Bidens cernua* and *Alisma plantago-aquatica*, were characteristic for NF and YF ponds, whereas LI and NM ponds contained relatively more late successional species, such as *Alnus glutinosa*, *Salix caprea* and J*uncus effusus*. Typical pioneering species were associated with high frequency of pond drainage. see Table S3 for a classification of emergent plants by Grime’s CSR strategy).

The first axis of the PCA ordination plot of mollusc community explained 31.5% of compositional variation and differentiated the NF management from other management types (Fig. 2C). The second axis comprised less variation (16.9%) and differentiated the NM management from other pond management types. Overall, most species were negatively associated with the NM management, while small bodied species, such *Anisus leucostomus*, *Anisus vortex,* *Segmentina nitida*, *Gyraulus crista* and *Gyraulus albus* showed a positive association with the NF management type. The majority of species was positively associated with high frequency of pond drainage.

The NF and YF management differed from the LI and NM management in hemipteran community composition (Fig. 2D). With the exception of *Sigara striata*, all Sigara species showed a clear positive association with NF and YF ponds, whereas larger species such as *Ranatra linearis*, *Naucoris maculatus* and *Notonecta glauca* tended to be more abundant in LI and NM ponds. In addition, most Sigara species were positively associated with drainage frequency and showed a negative association with the first summary variable of fish community composition.

The first axis of the PCA ordination plot of whole macro-invertebrate community comprised most compositional variation (26.9%) and differentiated the NF from the LI management (Fig. 2E). YF and NM ponds took an intermediate position on the plot. Most Diptera taxa were positively associated with LI ponds, whereas the majority of other taxa showed a preference for NF ponds. Forward selection revealed that pond surface and percentage of shore line covered with reed vegetation were the main environmental drivers for variation in macro-invertebrate community composition. Many taxa of Diptera tended to be positively associated with pond surface, while other macro-invertebrates were positively associated with reed cover and drainage frequency. With the exception of Diptera, most taxa showed a clear negative association with fish community biomass.

The majority of zooplankton species were positively associated with NF ponds and to a lesser extent also with NM ponds (Fig. 2F). NF ponds were characterized by a considerable fraction of macrophyte associated cladoceran species, such as *Simocephalus vetulus* and several *Ceriodaphnia* species.
