# Supplementary material for: The Impact of Conservation Management on the Community Composition of Multiple Organism Groups in Eutrophic Interconnected Man-Made Ponds
Source: PLoS One. 2015 Sep 30;10(9):e0139371. doi: 10.1371/journal.pone.0139371 (PMC4589289; doi:10.1371/journal.pone.0139371)
Supplement: S2 File — (DOCX) [file pone.0139371.s004.docx]

**S2 File. A detailed description of the pathways through which pond management type affected the investigated organism groups**

Our results also suggest that pond management affected different organism groups through different pathways. Pond management type explained approximately 5% of the compositional variation in submerged macrophyte community through the pond environment (Fig. S1B), whereas effects of management type through drainage were important for emergent plants and mollusks (R^2^_adj._ = 2.74% and 7.44%, respectively) (Fig. S1C and E). The ways through which management affected macro-invertebrate community composition were rather intricate (Fig. S1D). A relatively large amount of community variation was explained by management type in concert with effects on the fish communities and the pond environment (R^2^_adj._ = 6.12%). Furthermore, additional amounts of macro-invertebrate compositional variation was explained by the management-associated drainage frequency directly (R^2^_adj._ = 5.50%) and indirectly through the effect of drainage on fish community and local environmental conditions (R^2^_adj._ = 2.45%). Effects of management type on compositional variation in macro-invertebrate community through fish accounted for 2.18% of explained variation. Management type affected hemipteran communities indirectly through its effect on drainage frequency (R^2^_adj._ = 3.38%) and its effect on fish community via pond drainage (R^2^_adj._ = 1.81%) (Fig. S1F). The effect of management type solely via the fish community tended to be small (R^2^_adj._ = 1.09%). Pond management type affected zooplankton communities mainly indirectly through its effect on fish community characteristics (R^2^_adj._ = 4.70%) (Fig. S1G).
