# Supplementary material for: The Impact of Conservation Management on the Community Composition of Multiple Organism Groups in Eutrophic Interconnected Man-Made Ponds
Source: PLoS One. 2015 Sep 30;10(9):e0139371. doi: 10.1371/journal.pone.0139371 (PMC4589289; doi:10.1371/journal.pone.0139371)
Supplement: S1 Table — MAN = pond management type, FISH = fish community, ENV = local pond environment, DRAIN = frequency of pond drainage. PP = phytoplankton, SUBM = submerged and floating plants, EMERG = emergent plants, MOLL = mollusks, HEMI = hemipterans, MI = macro-invertebrates, ZP = zooplankton. Note that no variation partitioning analysis was done for phytoplankton since MAN, ENV, FISH nor DRAIN had an overall significant effect. Abundance data of organism groups were Hellinger transformed. (DOCX) [file pone.0139371.s005.docx]

**S1 Table. Detailed overview of the variables that were used in each statistical analysis**. MAN= pond management type, FISH = fish community, ENV= local pond environment, DRAIN= frequency of pond drainage. PP = phytoplankton, SUBM = submerged and floating plants, EMERG = emergent plants, MOLL = mollusks, HEMI = hemipterans, MI= macro-invertebrates, ZP = zooplankton. Note that no variation partitioning analysis was done for phytoplankton since MAN, ENV, FISH nor DRAIN had an overall significant effect. Abundance data of organism groups were Hellinger transformed.

|  |  | Explanatory variables | Response variables |
| --- | --- | --- | --- |
| *1. RDA to evaluate the effect of pond management type* | | |  |
|  | on target organism groups | MAN | community data of target organism groups |
|  | on fish | MAN | fish community data (biomass) |
|  | on pond environment | MAN | all local environmental pond variables |
|  |  |  |  |
| *2. RDA to evaluate the effect FISH, ENV and DRAIN on target organism groups* | | |  |
|  | effect of FISH | FISH (sample scores of PCA axis 1 and PCA axis 2) | community data of target organism groups |
|  | effect of ENV | ENV (all local environmental pond variables) | community data of target organism groups |
|  | effect of DRAIN | DRAIN | community data of target organism groups |
|  |  |  |  |
| *3+4. Variation Partitioning Analyses* | |  |  |
|  | on PP | - | - |
|  | on SUBM | MAN, ENV (pond surface area, conductivity and chl *a* concentration) *^1, 2^ | community data of SUBM |
|  | on EMERG | MAN, DRAIN *^1^ | community data of EMERG |
|  | on MOLL | MAN, DRAIN *^1^ | community data of MOLL |
|  | on HEMI | MAN, FISH (PCA axis 1, PCA axis 2), DRAIN *^2^ | community data of HEMI |
|  | on MI | MAN, FISH (PCA axis 1, PCA axis 2), ENV (pond surface area, coverage with reed), DRAIN | community data of MI |
|  | on ZP | MAN, FISH (sample scores of PCA axis 1 and PCA axis 2) *^2, 3^ | community data of ZP |
|  |  |  |  |
|  | *^1^ separate RDA analysis revealed that FISH had no significant effect | |  |
|  | *^2^ separate RDA analysis revealed that ENV had no significant effect | |  |
|  | *^3^ separate RDA analysis revealed that DRAIN had no significant effect | |  |
