# Supplementary material for: The Impact of Conservation Management on the Community Composition of Multiple Organism Groups in Eutrophic Interconnected Man-Made Ponds
Source: PLoS One. 2015 Sep 30;10(9):e0139371. doi: 10.1371/journal.pone.0139371 (PMC4589289; doi:10.1371/journal.pone.0139371)
Supplement: S2 Table — (DOCX) [file pone.0139371.s006.docx]

**S2 Table. Results from Spearman correlations between significant explanatory variables included in the RDA analyses for the different organism groups.**

| compared variables | R | p-values |
| --- | --- | --- |
| chla - surface area | -0.038 | 0.418 |
| chla -conductivity | -0.12 | 0.914 |
| conductivity - pond surface | 0.24 | 0.077 |
| Fish PCA 1 - Fish PCA 2 | 0.0062 | 0.909 |
| Fish PCA 1 - reed | -0.58 | **0.006** |
| Fish PCA 1 - drainage | -0.46 | **0.005** |
| Fish PCA 1 - surface area | 0.043 | 0.883 |
| Fish PCA 2 - reed | -0.022 | 0.725 |
| Fish PCA 2 - drainage | 0.041 | 0.451 |
| Fish PCA 2 - surface area | 0.29 | 0.115 |
