# Supplementary material for: The Impact of Conservation Management on the Community Composition of Multiple Organism Groups in Eutrophic Interconnected Man-Made Ponds
Source: PLoS One. 2015 Sep 30;10(9):e0139371. doi: 10.1371/journal.pone.0139371 (PMC4589289; doi:10.1371/journal.pone.0139371)
Supplement: S3 Table — Species with abbreviations are shown on the PCA ordination plots (Fig 2). Emergent plants were classified based on the CSR strategy. Classifications of the CSR strategy between parentheses were derived from knowledge of the authors. The Grime’s CSR classification of plants essentially classifies plant species according to the three trade-off strategies for survival: competitor (C), stress tolerant (S) and ruderal (R). These strategies each thrive best in a combination of either high or low intensity of stress and disturbance. (DOCX) [file pone.0139371.s007.docx]

**S3 Table. Overview of all taxa our dataset and their occurrence in each management type (NF= No Fish, YF = Young of the Year Fish, NM = No Management, LI = Low Intensity Management).** Species with abbreviations are shown on the PCA ordination plots (Fig. 2). Emergent plants were classified based on the CSR strategy [[1](#_ENREF_1)] according to Hodgson et al. [[2](#_ENREF_2)]. Classifications of the CSR strategy between parentheses were derived from knowledge of the authors. The Grime’s CSR classification of plants essentially classifies plant species according to the three trade-off strategies for survival: competitor (C), stress tolerant (S) and ruderal (R). These strategies each thrive best in a combination of either high or low intensity of stress and disturbance.

| Taxon name | abbreviation | CSR | NF | YF | NM | LI |
| --- | --- | --- | --- | --- | --- | --- |
| **Phytoplankton (genus name)** | | | | | | |
| Aulacoseira |  |  | 0 | 1 | 1 | 1 |
| Cyclotella |  |  | 1 | 1 | 0 | 1 |
| Nitzschia |  |  | 0 | 1 | 0 | 0 |
| pennaatspp. |  |  | 1 | 1 | 1 | 1 |
| Stephanodiscus |  |  | 0 | 1 | 1 | 1 |
| Synedra |  |  | 0 | 1 | 1 | 0 |
| Actinastrum |  |  | 0 | 1 | 1 | 1 |
| Ankistrodesmus |  |  | 1 | 1 | 1 | 0 |
| Ankyra |  |  | 0 | 1 | 0 | 0 |
| Botryococcus |  |  | 0 | 0 | 1 | 0 |
| Chlamydomonas |  |  | 1 | 0 | 1 | 0 |
| Chlorogonium |  |  | 1 | 1 | 0 | 0 |
| Closterium |  |  | 1 | 1 | 1 | 0 |
| coccaalsp. |  |  | 1 | 1 | 0 | 1 |
| Coelastrum |  |  | 0 | 1 | 1 | 1 |
| Cosmarium |  |  | 1 | 1 | 0 | 0 |
| Crucigenia |  |  | 1 | 1 | 1 | 1 |
| Desmidium |  |  | 1 | 0 | 0 | 0 |
| Desmodesmus |  |  | 1 | 1 | 1 | 1 |
| Dictyosphaerium |  |  | 1 | 1 | 1 | 1 |
| Euastrum |  |  | 0 | 1 | 0 | 0 |
| Eudorina |  |  | 1 | 1 | 0 | 1 |
| Golenkinia |  |  | 0 | 1 | 1 | 1 |
| Gonium |  |  | 0 | 1 | 1 | 0 |
| Kirchneriella |  |  | 1 | 1 | 1 | 1 |
| Koliella |  |  | 0 | 0 | 1 | 0 |
| Micractinium |  |  | 1 | 0 | 0 | 0 |
| Monoraphidium |  |  | 1 | 1 | 1 | 1 |
| Mougeotia |  |  | 1 | 1 | 1 | 1 |
| Oocystis |  |  | 1 | 0 | 1 | 1 |
| Pandorina |  |  | 1 | 1 | 1 | 1 |
| Pediastrum |  |  | 1 | 1 | 1 | 1 |
| Scenedesmus |  |  | 1 | 1 | 1 | 1 |
| Selenastrum |  |  | 1 | 1 | 1 | 1 |
| Sphaerellopsis |  |  | 0 | 0 | 1 | 0 |
| Staurastrum |  |  | 1 | 0 | 1 | 0 |
| Staurodesmus |  |  | 0 | 1 | 0 | 1 |
| Tetraedron |  |  | 1 | 1 | 1 | 1 |
| Tetrastrum |  |  | 1 | 0 | 1 | 0 |
| Tetrallantas |  |  | 1 | 0 | 0 | 0 |
| Treubaria |  |  | 0 | 0 | 0 | 1 |
| Ulothrix |  |  | 0 | 1 | 0 | 0 |
| Zygnema |  |  | 1 | 0 | 0 | 0 |
| cf.Phacotus |  |  | 1 | 1 | 0 | 0 |
| Gonyostomumsemen |  |  | 0 | 0 | 1 | 1 |
| Chrysococcus |  |  | 0 | 1 | 0 | 0 |
| Dinobryon |  |  | 0 | 0 | 1 | 0 |
| Mallomonas |  |  | 0 | 1 | 1 | 1 |
| Synura |  |  | 0 | 1 | 1 | 0 |
| Uroglena |  |  | 1 | 0 | 0 | 0 |
| Chroomonas |  |  | 0 | 1 | 0 | 0 |
| Cryptomonas |  |  | 1 | 1 | 1 | 1 |
| Katablepharis |  |  | 1 | 0 | 0 | 0 |
| Rhodomonas |  |  | 1 | 1 | 1 | 1 |
| Anabaena |  |  | 1 | 1 | 1 | 1 |
| Anabaenopsis |  |  | 1 | 1 | 1 | 0 |
| Aphanizomenon |  |  | 0 | 1 | 1 | 0 |
| Aphanocapsa |  |  | 1 | 1 | 1 | 1 |
| Geitlerinema |  |  | 0 | 1 | 1 | 1 |
| Limnothrix |  |  | 1 | 0 | 0 | 0 |
| Merismopedia |  |  | 1 | 1 | 1 | 1 |
| Microcystis |  |  | 0 | 1 | 1 | 1 |
| Oscillatoria |  |  | 0 | 0 | 0 | 1 |
| Phormidium |  |  | 0 | 1 | 0 | 0 |
| Pseudanabaena |  |  | 1 | 1 | 1 | 0 |
| Rhabdoderma |  |  | 0 | 1 | 0 | 0 |
| Spirulina |  |  | 0 | 1 | 0 | 0 |
| Ceratium |  |  | 0 | 1 | 1 | 1 |
| Gymnodinium |  |  | 1 | 1 | 1 | 1 |
| Peridinium |  |  | 1 | 1 | 1 | 1 |
| Euglena |  |  | 1 | 1 | 1 | 1 |
| Phacus |  |  | 1 | 1 | 1 | 1 |
| Strombomonas |  |  | 0 | 0 | 1 | 0 |
| Trachelomonas |  |  | 1 | 1 | 1 | 1 |
| Centritractus |  |  | 0 | 1 | 1 | 1 |
| Goniochloris |  |  | 1 | 1 | 1 | 1 |
| Pseudostaurastrum |  |  | 0 | 1 | 1 | 1 |
|  |  |  |  |  |  |  |
| **Submerged and floating plants (species name)** | | | | | | |
| *Apium nodiflorum* | *A. nodiflorum* |  | 1 | 0 | 0 | 0 |
| *Azolla filiculoides* | *A. filiculoides* |  | 0 | 1 | 0 | 0 |
| *Callitriche brutia* | *C. brutia* |  | 1 | 1 | 1 | 0 |
| *Callitriche hamalata* | *C. hamalata* |  | 1 | 1 | 0 | 0 |
| *Callitriche obtusangula* | *C. platycarpa* |  | 1 | 1 | 0 | 0 |
| *Callitriche palustris* |  |  | 0 | 1 | 0 | 0 |
| *Callitriche platycarpa* |  |  | 0 | 0 | 0 | 1 |
| *Callitriche sp.* |  |  | 1 | 0 | 0 | 0 |
| *Callitriche stagnalis* |  |  | 0 | 0 | 1 | 0 |
| *Ceratophyllum demersum* | *C. demersum* |  | 0 | 0 | 0 | 1 |
| *Chara brownii* | *C. brownii* |  | 1 | 0 | 0 | 0 |
| *Elatine hexandra* | *E. hexandra* |  | 1 | 1 | 1 | 0 |
| *Elatine hydropiper* |  |  | 1 | 1 | 1 | 1 |
| *Elatine sp.* |  |  | 0 | 1 | 0 | 0 |
| *Elatine triandra* |  |  | 1 | 1 | 0 | 1 |
| *Eleocharis acicularis* | *E. acicularis* |  | 1 | 1 | 1 | 0 |
| *Hydrocotyle vulgaris* | *H. vulgaris* |  | 1 | 0 | 1 | 0 |
| *Lemna minor* | *L. minor* |  | 1 | 1 | 1 | 1 |
| *Luronium natans* |  |  | 1 | 0 | 0 | 0 |
| *Lythrum portula* |  |  | 1 | 1 | 0 | 0 |
| *Najas marina* |  |  | 1 | 1 | 1 | 0 |
| *Nitella sp.* | *Nitella sp.* |  | 1 | 1 | 0 | 0 |
| *Nymphaea alba* | *N. alba* |  | 0 | 0 | 1 | 0 |
| *Persicaria amphibia* |  |  | 1 | 1 | 1 | 1 |
| *Potamogetaon trichoides* | *P. trichoides* |  | 1 | 0 | 0 | 0 |
| *Potamogeteon berchtoldii* | *P. berchtoldii* |  | 0 | 1 | 0 | 0 |
| *Potamogeton friessi* | *P. frissi* |  | 0 | 1 | 0 | 0 |
| *Potamogeton natans* |  |  | 1 | 1 | 1 | 0 |
| *Potamogeton obtusifolius* | *P. obtusifolius* |  | 1 | 1 | 1 | 0 |
| *Potamogeton pectinatus* |  |  | 1 | 1 | 0 | 0 |
| *Ranunculus aquatilis* |  |  | 1 | 1 | 0 | 0 |
| *Ranunculus pelatus* |  |  | 1 | 0 | 0 | 0 |
| *Riccia fluitans* | *R. fluitans* |  | 1 | 0 | 1 | 0 |
| *Sagittaria sagittifolio* |  |  | 0 | 1 | 1 | 0 |
| *Spirodela polyrhiza* |  |  | 0 | 1 | 0 | 0 |
| *Utricularia australis* |  |  | 1 | 0 | 1 | 0 |
| *Utricularia ochroleuca* |  |  | 0 | 0 | 1 | 0 |
| *Zannichellia palustris* |  |  | 0 | 1 | 0 | 0 |
|  |  |  |  |  |  |  |
| **Emergent plants (species name)** | | | | | | |
| *Agrostis stolonifera* |  | CR | 1 | 1 | 0 | 0 |
| *Alisma lanceolata* |  | R/CR | 0 | 1 | 1 | 0 |
| *Alisma plantago-aquatica* | *A. plantago-aquatica* | R/CR | 1 | 1 | 1 | 1 |
| *alnus glutinosa* | *A. glutinosa* | SC | 1 | 0 | 1 | 1 |
| *Alopecurus geniculatus* |  | CR | 0 | 1 | 0 | 0 |
| *Baldellia ranunculoides* |  | R/CR | 1 | 0 | 0 | 0 |
| *Beldellia repens* |  | (RS) | 1 | 0 | 0 | 0 |
| *Bidens cernua* | *B. cernua* | CR | 1 | 1 | 1 | 0 |
| *Bidens frontosa* | *B. frontosa* | (CR) | 1 | 1 | 1 | 1 |
| *Bidens tripartita* |  | CR | 0 | 0 | 1 | 0 |
| *Bolboschoenus maritimus* |  | C/SC | 0 | 1 | 1 | 1 |
| *Calamagrostis canescens* |  | C/SC | 0 | 0 | 1 | 0 |
| *Calamgrostis epigejos* |  | C/SC | 1 | 0 | 1 | 0 |
| *Carex acuta* |  | C/SC | 1 | 0 | 0 | 1 |
| *Carex acutiformis* | *C. acutiformis* | C/SC | 1 | 0 | 1 | 0 |
| *Carex disticha* |  | C/CSR | 0 | 0 | 1 | 0 |
| *Carex pseudocyperus* | *C. pseudocyperus* | C/CSR | 1 | 1 | 1 | 0 |
| *Carex riparia* | *C. riparia* | C/SC | 1 | 0 | 1 | 1 |
| *Carex rostrata* |  | SC | 0 | 0 | 1 | 0 |
| *Cyperus fuscus* |  | (RS) | 0 | 1 | 0 | 0 |
| *Eleocharis multicaulis* |  | SC/CSR | 1 | 0 | 0 | 0 |
| *Eleocharis palustris* | *E. palustris* | C/CSR | 1 | 1 | 1 | 1 |
| *Epilobium hirsutum* |  | C | 0 | 1 | 0 | 0 |
| *Epilobium parviflorum* | *E. parviflorum* | CSR | 0 | 0 | 0 | 1 |
| *Festuca arundinacea* | *F. arundinacea* | SC/CSR | 0 | 1 | 0 | 0 |
| *Galium palustre* |  | CR/CSR | 1 | 0 | 0 | 0 |
| *Galium ulginosum* | *G. ulginosum* | CSR | 0 | 0 | 1 | 0 |
| *Glyceria maxima* |  | C | 1 | 0 | 0 | 0 |
| *Glyceria notata* | *G. notata* | CR | 1 | 0 | 1 | 0 |
| *Glyceria sp.* |  | (CR/C) | 0 | 1 | 0 | 0 |
| *Iris pseudacorus* | *I. pseudacorus* | C/CSR | 1 | 0 | 1 | 1 |
| *Juncus articulatus* |  | CR/CSR | 1 | 0 | 0 | 0 |
| *Juncus bufonius* |  | R/SR | 0 | 0 | 1 | 0 |
| *Juncus bulbosus* |  | S/SR | 1 | 1 | 1 | 0 |
| *Juncus effusus* | *J. effusus* | C/SC | 1 | 1 | 1 | 1 |
| *Lindernia dubia* | *L. dubia* | (R) | 1 | 1 | 1 | 1 |
| *Litorella uniflora* |  | SR/CSR | 1 | 0 | 0 | 0 |
| *Lotus pendunculatus* |  | C/CSR | 0 | 0 | 1 | 0 |
| *Ludwigia palustris* |  | (RS) | 1 | 1 | 1 | 1 |
| *Lycopus europaeus* |  | C/CR | 1 | 1 | 1 | 1 |
| *Lysimachia vulgaris* | *L. vulgaris* | C | 1 | 1 | 1 | 1 |
| *Lythrum salicaria* | *L. salicaria* | C/CR | 1 | 1 | 1 | 1 |
| *Mentha aquatica* | *M. caerulea* | C/CR | 1 | 1 | 0 | 0 |
| *Molinia caerulea* |  | SC | 0 | 0 | 1 | 0 |
| *Persicaria hydropiper* |  | R/CR | 1 | 1 | 1 | 1 |
| *Persicaria lapathifolia* | *P. lapathifolia* | CR | 0 | 1 | 1 | 0 |
| *Persicaria maculosa* | *P. maculosa* | R/CR | 0 | 1 | 0 | 0 |
| *Persicaria minor* | *P. minor* | R/SR | 1 | 1 | 0 | 0 |
| *Persicaria mitis* |  | (R/SR) | 0 | 1 | 0 | 0 |
| *Peucedanum palustre* | *P. palustre* | (C/CR) | 0 | 0 | 1 | 1 |
| *Phalaris arundinacea* |  | C | 0 | 0 | 1 | 0 |
| *Phramites australis* | *P. australis* | C | 1 | 1 | 1 | 1 |
| *Pilularia globulifera* |  | R/SR | 1 | 0 | 0 | 0 |
| *Pseucedanum palustre* |  | (SR) | 0 | 1 | 0 | 0 |
| *Ranunculus flammula* |  | CR/CSR | 1 | 0 | 0 | 0 |
| *Ranunculus repens* | *R. repens* | CR | 0 | 1 | 0 | 0 |
| *Ranunculus scleratus* | *R. scleratus* | R/CR | 0 | 1 | 0 | 1 |
| *Rumex hydrolapathum* | *R. hydrolapathum* | C/CSR | 1 | 0 | 1 | 1 |
| *Rumex maritimus* | *R. maritimus* | R/CR | 0 | 1 | 0 | 0 |
| *Rumex palustris* |  | (R/CR) | 0 | 1 | 1 | 0 |
| *Salix caprea* | *S. caprea* | C/SC | 1 | 0 | 1 | 1 |
| *Salix sp.* |  | C/SC | 0 | 1 | 1 | 0 |
| *Schoenoplectus lacustris* | *S. lacustris* | C/SC | 1 | 1 | 0 | 1 |
| *Schoenoplectus tabernaemontani* | *S. tabernaemontani* | C/SC | 1 | 1 | 1 | 1 |
| *Scirpus sylvaticus* | *S. galericulata* | C | 1 | 0 | 0 | 1 |
| *Scutellaria galericulata* |  | CR/CSR | 1 | 0 | 0 | 1 |
| *Senecio inaequidens* |  | (CR) | 0 | 1 | 0 | 0 |
| *Solanum dulcamare* |  | C/CSR | 0 | 0 | 1 | 0 |
| *Sparganium emersum* |  | CR | 1 | 0 | 1 | 0 |
| *Sparganium erectum* | *S. erectum* | C/CR | 1 | 0 | 0 | 0 |
| *Stachys palustris* | *S.palustris* | C/CR | 0 | 1 | 0 | 1 |
| *Typha angustifolia* | *T. angustifolia* | C/SC | 1 | 0 | 1 | 0 |
| *Typha latifolia* | *T.latifolia* | C | 1 | 1 | 1 | 1 |
|  |  |  |  |  |  |  |
| **Zooplankton (species name, except copepods)** | | | | | | |
| *Acroperus harpae* |  |  | 0 | 1 | 1 | 0 |
| *Alona costata* |  |  | 1 | 1 | 0 | 0 |
| *Alona guttata* |  |  | 0 | 1 | 1 | 1 |
| *Alona quadrangularis* |  |  | 0 | 0 | 1 | 0 |
| *Alona rectangula* |  |  | 1 | 1 | 1 | 1 |
| *Alonella exigua* |  |  | 1 | 0 | 0 | 0 |
| *Alonella nana* |  |  | 1 | 0 | 1 | 0 |
| *Bosmina coregoni* |  |  | 0 | 1 | 1 | 1 |
| *Camptocercus rectirostris* |  |  | 0 | 0 | 1 | 0 |
| *Ceriodaphnia dubia* | *C. dubia* |  | 1 | 0 | 0 | 0 |
| *Ceriodaphnia laticaudata* |  |  | 0 | 1 | 0 | 1 |
| *Ceriodaphnia megops* | *C. megops* |  | 1 | 0 | 0 | 0 |
| *Ceriodaphnia pulchella* |  |  | 1 | 1 | 1 | 1 |
| *Ceriodaphnia quadrangula* | *C. quadrangula* |  | 1 | 1 | 1 | 0 |
| *Ceriodaphnia reticulata* | *C. reticulata* |  | 1 | 0 | 1 | 0 |
| *Chydorus sphaericus* |  |  | 1 | 1 | 1 | 1 |
| *Daphnia cucullata* | *D. complex* |  | 0 | 0 | 1 | 0 |
| *Daphnia hyalina* |  |  | 1 | 1 | 0 | 1 |
| *Daphnia longispina* | *D. longispina* |  | 1 | 0 | 0 | 1 |
| *Daphnia magna* | *D. magna* |  | 0 | 1 | 0 | 0 |
| *Daphnia obtusa* | *D. obtusa* |  | 0 | 0 | 0 | 1 |
| *Daphnia pulex* |  |  | 0 | 1 | 0 | 0 |
| *Diaphanosoma brachyurum* |  |  | 1 | 1 | 1 | 1 |
| *Eurycercus lamellatus* | *E. lamellatus* |  | 1 | 1 | 1 | 0 |
| *Graptoleberis testudinaria* | *G. testudinaria* |  | 1 | 0 | 1 | 0 |
| *Iliocryptus sordidus* |  |  | 0 | 1 | 1 | 0 |
| *Kurzia latissima* | *K. latissima* |  | 1 | 0 | 0 | 0 |
| *Leptodora kindtii* |  |  | 0 | 0 | 1 | 0 |
| *Leydigia leydigi* |  |  | 0 | 0 | 1 | 1 |
| *Macrothrix rosea* | *M. rosea* |  | 1 | 0 | 0 | 0 |
| *Megafenestra aurita* | *M. aurita* |  | 1 | 1 | 0 | 0 |
| *Moina brachiata* |  |  | 0 | 1 | 0 | 1 |
| *Moina macrocopa* |  |  | 0 | 1 | 1 | 0 |
| *Pleuroxus denticulatus* | *P. denticulatus* |  | 1 | 0 | 1 | 1 |
| *Pleuroxus aduncus* |  |  | 1 | 1 | 1 | 1 |
| *Pleuroxus truncatus* |  |  | 0 | 0 | 1 | 1 |
| *Pleuroxus unicatus* | *P. unicatus* |  | 0 | 0 | 1 | 0 |
| *Polyphemus pediculus* |  |  | 1 | 0 | 0 | 0 |
| *Scapholeberis mucronata* |  |  | 1 | 1 | 1 | 1 |
| *Scapholeberis rammneri* |  |  | 1 | 1 | 0 | 0 |
| *Sida crystallina* |  |  | 0 | 0 | 1 | 1 |
| *Simocephalus exspinosus* | *S. exspinosus* |  | 0 | 0 | 1 | 0 |
| *Simocephalus serrulatus* | *S. serrulatus* |  | 1 | 0 | 0 | 0 |
| *Simocephalus vetulus* | *S. vetulus* |  | 1 | 1 | 1 | 1 |
| *Cyclopoida (cyclopoid copepods)* |  |  | 1 | 1 | 1 | 1 |
| *Calanoida (calanoid copepods)* |  |  | 1 | 1 | 1 | 1 |
|  |  |  |  |  |  |  |
| **Molluks (species name)** | | | | | | |
| *Acroloxus lacustris* | *A. lacustris* |  | 1 | 0 | 1 | 0 |
| *Anisus leucostomus* | *A. leucostomus* |  | 1 | 0 | 0 | 0 |
| *Anisus vortex* | *A. vortex* |  | 1 | 0 | 0 | 0 |
| *Bathyomphales contortus* | *B. contortus* |  | 0 | 0 | 1 | 0 |
| *Gyraulus albus* | *G. albus* |  | 1 | 1 | 1 | 1 |
| *Gyraulus crista* | *G. crista* |  | 1 | 1 | 1 | 0 |
| *Gyraulus laevis* | *G. laevis* |  | 1 | 1 | 1 | 1 |
| *Hippeutis complanatus* | *H. complanatus* |  | 0 | 0 | 1 | 0 |
| *Lymnaea auricularia* | *L. auricularia* |  | 1 | 1 | 1 | 0 |
| *Lymnaea glabra* | *L. glabra* |  | 0 | 1 | 0 | 0 |
| *Lymnaea peregra* | *L. peregra* |  | 1 | 1 | 1 | 1 |
| *Lymnaea stagnalis* | *L. stagnalis* |  | 1 | 1 | 1 | 1 |
| *Lymnaea trunculata* | *L. trunculata* |  | 1 | 1 | 0 | 0 |
| *Physa acuta* | *P. acuta* |  | 1 | 1 | 1 | 1 |
| *Planorbarius corneus* | *P. corneus* |  | 1 | 1 | 1 | 1 |
| *Planorbis caenatis* | *P. carinatus* |  | 1 | 0 | 0 | 1 |
| *Planorbis planorbis* | *P. planorbis* |  | 1 | 1 | 1 | 0 |
| *Segmentina nitida* | *S. nitida* |  | 1 | 0 | 0 | 0 |
| *Sphaerum corneum* | *S. corneum* |  | 1 | 1 | 1 | 1 |
| *Viviparus contectus* | *V. contectus* |  | 0 | 0 | 1 | 0 |
|  |  |  |  |  |  |  |
| **Hemiptera (species name)** | | | | | | |
| *Callicorixa praeusta* | *C. praeusta* |  | 1 | 1 | 1 | 1 |
| *Corixa punctata* | *C. punctata* |  | 1 | 1 | 1 | 1 |
| *Cymatia bonsdorfii* | *C. bonsdorfii* |  | 1 | 0 | 0 | 0 |
| *Cymatia coleoptrata* | *C. coleoptrata* |  | 1 | 1 | 1 | 0 |
| *Hesperocorixa castanae* | *H. castanae* |  | 1 | 0 | 0 | 0 |
| *Hesperocorixa linnei* | *H. linnei* |  | 1 | 0 | 1 | 0 |
| *Micronecta sp.* | *Micronecta sp.* |  | 1 | 0 | 0 | 0 |
| *Naucoris maculatis* | *N. maculatis* |  | 1 | 0 | 1 | 1 |
| *Notonecta glauca* | *N. glauca* |  | 1 | 0 | 1 | 0 |
| *Pleia minutissima* | *P. minutissima* |  | 1 | 0 | 1 | 0 |
| *Ranata linearis* | *R. linearis* |  | 0 | 0 | 1 | 0 |
| *Sigara distincta* | *S. distincta* |  | 1 | 1 | 1 | 1 |
| *Sigara falleni* | *S. falleni* |  | 1 | 1 | 1 | 1 |
| *Sigara lateralis* | *S. lateralis* |  | 1 | 1 | 0 | 1 |
| *Sigara semistriata* | *S. semistriata* |  | 1 | 1 | 0 | 0 |
| *Sigara striata* | *S. striata* |  | 1 | 1 | 1 | 1 |
|  |  |  |  |  |  |  |
| **Overall macro-invertebrates (family name, except Lepidoptera and Hirundinea)** | | | | | |  |
| Acroloxidae |  |  | 1 | 0 | 1 | 0 |
| Asellidae |  |  | 1 | 1 | 1 | 1 |
| Baetidae |  |  | 1 | 1 | 1 | 1 |
| Bithyniidae |  |  | 0 | 0 | 0 | 0 |
| Caenidae |  |  | 1 | 1 | 1 | 1 |
| Ceratopogonidae |  |  | 1 | 1 | 1 | 1 |
| Chaoboridae |  |  | 1 | 1 | 1 | 1 |
| Chironomidae |  |  | 1 | 1 | 1 | 1 |
| Corixidae |  |  | 1 | 1 | 1 | 1 |
| Culicidae |  |  | 1 | 1 | 1 | 1 |
| Cylindrotomidae |  |  | 0 | 0 | 0 | 1 |
| Dixidae |  |  | 1 | 1 | 1 | 1 |
| Empidae |  |  | 1 | 1 | 0 | 0 |
| Ephydridae |  |  | 1 | 1 | 0 | 0 |
| Gammaridae |  |  | 1 | 1 | 0 | 1 |
| Hirundinea |  |  | 1 | 1 | 1 | 1 |
| Lepidoptera |  |  | 1 | 1 | 1 | 0 |
| Limonidae |  |  | 1 | 1 | 1 | 1 |
| Lymnaeidae |  |  | 1 | 1 | 1 | 1 |
| Muscidae |  |  | 1 | 0 | 0 | 0 |
| Naucoridae |  |  | 1 | 0 | 1 | 1 |
| Nepidae |  |  | 0 | 0 | 1 | 0 |
| Notonectidae |  |  | 1 | 0 | 1 | 0 |
| Physidae |  |  | 1 | 1 | 1 | 1 |
| Planorbidae |  |  | 1 | 1 | 1 | 1 |
| Pleidae |  |  | 1 | 0 | 1 | 0 |
| Psychodidae |  |  | 0 | 1 | 0 | 0 |
| Ptychopteridae |  |  | 1 | 1 | 0 | 0 |
| Sciomizydae |  |  | 1 | 0 | 0 | 0 |
| Sphaeriidae |  |  | 1 | 1 | 1 | 1 |
| Stratiomydae |  |  | 1 | 1 | 1 | 1 |
| Valvatidae |  |  | 0 | 0 | 0 | 0 |
| Viviparidae |  |  | 0 | 0 | 1 | 0 |

**REFERENCES**

1. Grime J (1974) Vegetation classification by reference to strategies. Nature 250: 26-31.

2. Hodgson J, Wilson P, Hunt R, Grime J, Thompson K (1999) Allocating CSR plant functional types: a soft approach to a hard problem. Oikos 85: 282-294.
